# Four-dimensional velocity encoded MRI improves blood flow quantification in patients with semilunar valve stenosis

**DOI:** 10.1186/1532-429X-14-S1-W10

**Published:** 2012-02-01

**Authors:** Sarah Nordmeyer, Eugenie Riesenkampff, Daniel Messroghli, Felix Berger, Titus Kuehne

**Affiliations:** 1 Department of Congenital Heart Disease/Pediatric Cardiology, Deutsches Herzzentrum Berlin, Berlin, Germany

## Background

Stenoses might cause complex flow patterns, which are sometimes difficult to assess quantitatively with standard two-dimensional (2D) VEC MRI. We sought to evaluate the use of four-dimensional (4D) velocity encoded magnetic resonance imaging (VEC MRI) for blood flow quantification in patients with semilunar valve stenosis.

## Methods

Peak velocities (Vmax) and stroke volumes (SV) were quantified by 2D and 4D VEC MRI in volunteers (n=7) and patients with semilunar valve stenosis (n=18). Measurements were performed immediately above the aortic or pulmonary valve with both techniques (=level 1) and, additionally, at further predefined planes in the ascending aorta and in the pulmonary trunk within the 4D dataset. 4D VEC MRI streamline analysis identified “individualized” planes of highest flow velocity (4Dmax-targeted) for further measurements. In patients, Vmax was also measured by Doppler-echocardiography.

## Results

In patients, 4D VEC MRI (2.7m/s) showed comparable Vmax to Doppler-echocardiography (2.8m/s) and significantly higher Vmax than 2D VEC MRI (2.4m/s) (p<0.03) at level 1. 4Dmax-targeted revealed highest Vmax values (3.1m/s). Correlations of MR-derived peak velocities with Doppler-echocardiography were r=0.62 for 2D, r=0.67 for 4D at level 1 and r=0.80 for 4Dmax-targeted. 4D showed higher agreement with Doppler-echocardiography than did 2D VEC MRI at level 1 (p=0.039).

SV at level 1 were comparable between both techniques. SV measurements at different anatomical levels in the ascending aorta showed a significantly larger variance in patients with complex flow patterns than in volunteers with laminar flow patterns (p=0.004).

## Conclusions

4D VEC MRI improves MR-derived quantification of peak flow velocities in patients with semilunar valve stenosis. Additionally, 4D VEC MRI demonstrates the potential for SV underestimation in complex flow.

**Table 1:** Comparison between 2D (level 1) and 4D (level 1 and 4D max-targeted) derived peak velocity (+Doppler-echocardiography) in the ascending aorta in patients with aortic valve stenosis and in the pulmonary trunk in patients with pulmonary valve stenosis

Patients	Vmax (m/s) ECHO	Vmax (m/s) 2D Level 1	Vmax (m/s) 4D Level 1	Vmax (m/s) 4D max-targeted
Mean	2.78	2.40	2.74	3.12
SD	0.68	0.54	0.64	0.63
p-value*	0.009	-	0.025	< 0.001
p-value**	-	<0.01	>0.05	<0.05

Vmax = peak velocity, 2D = two-dimensional, 4D = four-dimensional, SD = standard deviation, Echo = Doppler-echocardiography, * = compared to 2D, ** = compared to Echo

**Figure 1:**
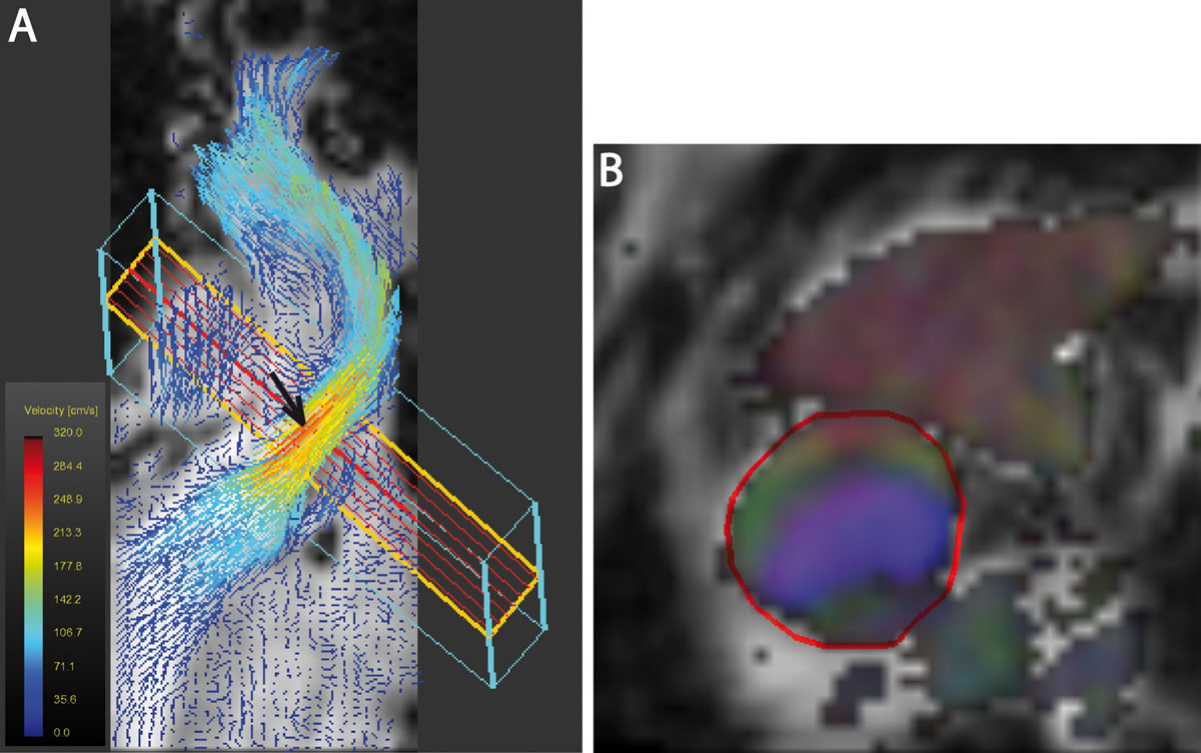
Assessment of peak velocity using flow visualization by 4D VEC MRI Figure: Velocity information in the left ventricular outflow tract of a patient with bicuspid aortic valve. A) Coronal axis of the left ventricular outflow tract, aortic valve and ascending aorta with in-plane visualization of velocity information. Dark red (black arrow) visualizes site of peak velocity. B) 4Dmax-targeted plane for blood flow quantification with colour coded flow direction.

